# Composition of pathogenic microorganism in chronic osteomyelitis based on metagenomic sequencing and its application value in etiological diagnosis

**DOI:** 10.1186/s12866-023-03046-x

**Published:** 2023-10-28

**Authors:** Kang Zhang, Yu-zhe Bai, Chang Liu, Shan-shan Liu, Xin-xin Lu, Run-gong Yang

**Affiliations:** 1 Laboratory Medicine of Beijing Tongren Hospital affiliated to Capital Medical University, Beijing, China; 2 Department of Tissue Repair and Regeneration, The First Medical Center of PLA General Hospital, Beijing, China; 3 Clinical Laboratory of Tsinghua University Hospital, Beijing, China

**Keywords:** Chronic osteomyelitis, Microorganism, mNGS, Culture method, Application value

## Abstract

**Background:**

Traditionally, conventional microbiological culture methods have been used to detect pathogenic microorganisms in chronic osteomyelitis. However, these methods have been found to have a low detection rate, complicating the precise guidance of infection treatment. This study employed metagenomic next-generation sequencing (mNGS) to detect these microorganisms in chronic osteomyelitis with three main objectives: 1). Gain a deeper understanding of the composition of pathogenic microorganisms in chronic osteomyelitis. 2). Compare the microbial detection rates between mNGS and the standard culture methods used in laboratories to enhance the effectiveness of the traditional culture methods. 3). Explore the potential of mNGS in etiological diagnosis.

**Methods:**

Fifty clinically confirmed intraoperative bone tissue samples of chronic osteomyelitis from January 2021 to December 2021 were collected and subjected to mNGS and microbiological testing, respectively. The orthopaedic surgeon combined clinical manifestations and related examinations to determine the causative pathogens.

**Results:**

The culture method obtained 29 aerobic and parthenogenic anaerobic bacteria, 3 specific anaerobic bacteria, and 1 yeast-like fungus. Thirty-six aerobic and parthenogenic anaerobic bacteria, 11 specific anaerobic bacteria, and 1 yeast-like fungus were obtained by mNGS, and 2 *Mycobacterium tuberculosis*(MTB) strains were detected. However, there was no significant difference in the overall positive detection rate between mNGS and the culture method (*P* = 0.07), and the two were not statistically significant in detecting aerobic and partly anaerobic bacteria (*P* = 0.625). But, mNGS was significantly superior to culture in detecting anaerobic bacteria and Mycobacterium tuberculosis (*P*<0.05).

**Conclusions:**

The mNGS method has enhanced our understanding of the distribution of pathogenic microorganisms in chronic osteomyelitis. Traditional culture methods help isolate and cultivate aerobic and facultative anaerobic bacteria, and fungi, and are also utilized for antibacterial drug sensitivity tests. However, mNGS has shown superior capabilities in detecting anaerobic bacteria, MTB, and mixed infection bacteria. This finding offers invaluable guidance for improving laboratory microbial culture and detection conditions. Hence, mNGS should be judiciously used for chronic osteomyelitis, and PCR can be implemented for certain difficult-to-culture microorganisms, such as MTB.

**Supplementary Information:**

The online version contains supplementary material available at 10.1186/s12866-023-03046-x.

## Introduction

Open fractures, infections of adjacent bone tissues, hematogenous infections, and infections after internal fracture fixation often cause chronic osteomyelitis. More so, it can involve single or multiple areas of the bone, such as the bone marrow, cortex, periosteum, or surrounding soft tissues [1]. Chronic osteomyelitis is a complex condition with a high treatment failure and recurrence rate, which some patients may face [2, 3]. The infection rate for open fractures and instrumented fixation in Europe and the United States is 5% [4]. In contrast, the infection rate after open fractures in some regions of China exceeds 30% [5, 6]. Additionally, the amputation rate in studies related to traumatic osteomyelitis of the extremities in South China was 3.71%, with an overall disability rate of 4.45% [7]and a recurrence rate of 12.36% in some patients [8]. Chronic osteomyelitis requires early identification of the pathogenic microorganism and targeted anti-infection before follow-up treatment. Pathogenic microbiological testing is still culture-based, with a positive rate of 40-85% [9]. However, it has a higher probability of negative results [10]due to the effects of antibiotics, low microbial load, difficult culture, and biofilm. Culture, identification, and drug sensitivity tests usually take 3–6 days or even 6 weeks. Therefore, the culture method has limitations for the pathogenic diagnosis of osteomyelitis [11].

Metagenomic next-generation sequencing (mNGS) is an unbiased, antibiotic-use-independent, rapid nucleic acid sequencing method for unknown or difficult-to-culture pathogenic microorganisms that can diagnose various infectious diseases [12–15]. Still, it is also a costly test, which most patients need help to afford. Mengchen Zou et al. [16] used mNGS in a study of the microbial composition of diabetic foot osteomyelitis, showing that it detected more microbial species than the culture method and confirming the feasibility of this technique for identifying microorganisms in diabetic foot osteomyelitis. Additionally, mNGS has been applied in the study of osteoarticular infections (OAI), and the authors concluded that mNGS is a reliable tool for detecting pathogenic microorganisms in OAI [11, 17]. The composition of pathogenic microorganisms in patients with chronic osteomyelitis remains uncertain. This study used mNGS to explore the distribution of pathogenic microorganisms in chronic osteomyelitis and to compare the differences in microbial distribution and detection rate with common culture methods. This dual approach suggests improvements for traditional laboratory culture methods and informs clinicians about the potential application of mNGS in etiological diagnosis. Furthermore, it is recommended that microbial culture be combined with nucleic acid detection for a more comprehensive diagnosis. The specifics of the research are outlined below.

## Methods

### Inclusion and exclusion criteria

Inclusion criteria: (i) disease duration > 1 month and clinical diagnosis of chronic osteomyelitis [18]; (ii) traumatic osteomyelitis with a history of traumatic fracture surgery of the extremities and clinical symptoms, signs and imaging manifestations of bone and soft tissue infection in the surgical area after surgery; (iii) patients with chronic hematogenous osteomyelitis who were ineffective with antibiotic treatment and required surgical treatment.

Exclusion criteria: (i) diabetic foot osteomyelitis; (ii) antibiotics used within 2 weeks before surgery.

### Study population

**Fig. 1:**
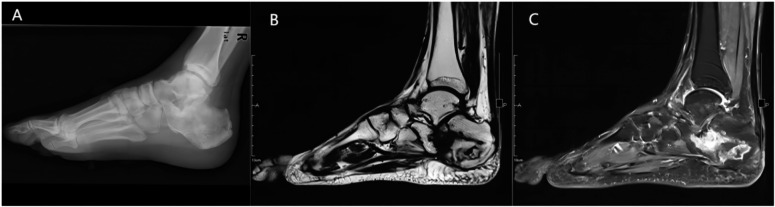
Imaging manifestations of diffuse osteomyelitis. **A**. X-ray showed an irregular arrangement of bone trabeculae and heterogeneous bone density. **B** and **C**. MRI showed a bone fracture in the heel bone, a lamellar low signal shadow in the heel bone on the T1-weighted image, and an irregular large lamellar high signal shadow in the heel bone on the T2 compression lipid image

Sixty-three samples from 55 cases of diffuse osteomyelitis (Fig. 1) with Cierne-Mader anatomical typing of type IV [19] admitted from January 2021 to December 2021 were collected. Eight samples from five patients were excluded due to obvious contamination and sequencing failure during sampling and pretreatment. All patients’ genders, ages, infection sites, imaging, pathological examination, and laboratory-related tests were recorded.

### Sample collection and preprocessing

The samples intraoperative retained bone with surrounding soft tissue and pus in the medullary cavity. Tissue in the operative area: 4 ~ 6 copies of cancellous bone and peripheral soft tissues from different regions of the same site were retained in each case, numbered according to the area taken, placed in sterile containers, pretreated separately for microbiological testing, and processed within 2 h. Samples for mNGS were placed directly in a special sampling tube with fluid (Nucleic acid removal) and transported to the laboratory on dry ice within 24 h. Bone and large tissue samples were placed in sterile saline and sonicated at ≥ 20 kHz for 7 min at room temperature [20]. After sonication, the liquid was centrifuged (3,200×g, 20 min) to discard the supernatant, and 2 ml of sterile saline was added. Small tissue specimens were ground into a homogenate using a mechanical grinder with 2 ml of sterile saline.

### Traditional microbiological testing

All samples were subjected to morphological examination with microbiological culture.

Morphological examination of the Gram, acid-fast, and weak acid-fast stain was performed according to the manufacturers’ (Zhuhai Baso Biotechnology Co., Ltd., China) instructions.

#### Common bacterial culture

After pretreatment, multiple samples were inoculated on Columbia blood plates and Roseola China Blue plates (Thermo Fisher Scientific, USA) and incubated at 35℃ 5% CO_2_ for 5 ~ 7 days.

#### Anaerobic culture

Inoculated in anaerobic phenyl ethanol medium (Guangzhou Dijing Bio, China), placed in anaerobic bags sealed with 3.5 L anaerobic gas production kit and anaerobic indicator (BioMérieux, France), and incubated at 35℃ 5% ambient for 7 days.

#### Fungal culture

Inoculated in Sabouraud’s weak medium (Thermo Fisher Scientific, USA), a small piece of tissue was spotted in the medium and incubated at 30 °C for 7 days.

#### Mycobacterial culture

Inoculated in Roche solid medium (Zhuhai Baso Biotechnology Co., Ltd., China) and incubated at 35℃ 5% CO_2_ for 4 ~ 6 weeks.

Microbial identification and drug sensitivity test:

Microbial identification was performed using Matrix-Assisted Laser Desorption/ Ionization Time of Flight Mass Spectrometry (MALDI-TOF MS, BioMérieux, France). The instrumental method for drug sensitivity testing was used with the VITEK 2-compact microbial drug sensitivity system (BioMérieux, France), and the E-test method (Zhengzhou Anto Bio) was chosen for the manual method. Yeast-like fungi were tested using the ATBTM FUNGUS3 (BioMérieux, France) microdilution method. Furthermore, the results of the drug sensitivity tests were interpreted according to the CLSI (Clinical and Laboratory Standards Institute) executive standard.

### mNGS Testing

The sample is placed in a special sampling tube and transported to WillingMed Technology (Beijing) Co., Ltd. for testing by the cold chain.

#### Sample collection and DNA extraction

DNA was extracted using PathoXtract® Basic Pathogen Nucleic Acid Kit (WYXM03211S, WillingMed company), and RNA was extracted using PathoXtract® Virus DNA/RNA Isolation Kit (WYXM03009S, WillingMed company) according to the manufacturer’s protocol. DNA and RNA were mixed, and then reverse transcription of the RNA to complementary DNA (cDNA) was performed using SuperScript® Double-Stranded cDNA Synthesis Kit (11,917,020, Invitrogen).

#### Library construction and sequencing

Libraries for NGS were prepared from mixed DNA using the Illumina® DNA Prep, (M) Tagmentation (20,018,705, Illumina) according to the manufacturer’s recommendation. Pooled libraries were sequenced on NextSeq™ 550Dx system using a 75 bp, single-end sequencing kit (Illumina), and at least 20 million sequencing reads were acquired for each sample.

#### Pipeline of bioinformatics analysis

The genomic data of bacteria, fungi, viruses, parasites, archaea, and other pathogenic microorganisms were obtained from NCBI GenBank, and the clinical application-level reference database of pathogenic microorganisms was constructed through genomic filtering, screening, and validation. Quality control and evaluation of FASTQ format data obtained by sequencing were carried out, and low-quality or undetected sequences, sequences contaminated by splices, high-coverage repeats, and short read-length sequences were filtered to retain high-quality sequencing data. The high-quality sequencing data were compared with the human reference genome GRCH37 (hg19) by alignment software to remove the human host sequence and obtain clean to identify pathogenic microorganisms.The clean data were aligned with the established reference database of pathogenic microorganisms to complete the annotation of pathogenic microorganism species, complete the final analysis, and obtain results on microorganism identification.

### Interpretation of results

Combining international guidelines on treating chronic osteomyelitis and referring to relevant research literature, we determined the criteria for microbial culture and mNGS. Finally, we determined the clinically significant pathogenic microorganisms by clinicians.

#### Laboratory diagnosis

1. Culture-positive judgment criteria: two or more out of four to six samples grew identical and phenotypically indistinguishable microorganisms [21].
2. mNGS judgment criteria: Sequencing labs exclude background microorganisms by self-constructed background databases and verify pre-defined thresholds using independent validation sets. The thresholds for positive microorganisms were determined concerning the Catalogue of Pathogenic Microorganisms of Human Transmission issued by the National Health and Wellness Commission of the People’s Republic of China and published supporting literature using the same sequencing platform [22].This was expressed as the number of sequence reads (RPTMs) that aligned to the genome of the target species per million sequences, specifically:

Bacteria ≥ 8 (RPTM);

Fungi ≥ 8 (RPTM);

MTB ≥ 1 (RPTM);

Virus ≥ 3 (RPTM).

#### Clinical composite diagnosis as the reference standard

The orthopaedic surgeon reviewed the patients’ medical records and microbiological tests (microscopy, culture, and mNGS) and identified the causative pathogens determined based on clinical findings, laboratory tests, radiology, pathology, and treatment observations.

### Statistical analysis

Statistical analysis was performed using SPSS statistical software (IBM Corporation, Armonk, NY, USA). Normally distributed measures were expressed as mean ± standard deviation, and non-normally distributed measures were expressed as median (M) and interquartile range (IQR). Statistical data were expressed as frequencies and percentages (%), and the chi-square test analyzed group comparisons. *P* < 0. 05 was considered a statistically significant difference.

## Results

### Patient clinical characteristics

There were 40 male and 10 female patients in 50 cases. The age distribution was 11–72 years, with a median of 52 years. The top three routes of infection were 31 cases of disease after internal fracture fixation, 6 cases of hematogenous osteomyelitis, and 6 cases of infection of bone adjacent tissues. The most common sites where osteomyelitis occurs are the tibia, femur, and heel bone. The disease duration ranged from 3 months to a maximum of 44 years. Notably, the most frequent comorbid underlying diseases were hypertension and diabetes mellitus. The clinical characteristics of the patients are shown in Supplemental File 1 and Table 1.

**Table 1:** Demographic information

Characteristic	Result
**Age (year), median (IQR)**	52, (34.25,62)
**Male, n(%)**	40(80%)
**Location**	
Tibia, n(%)	17(34%)
Femur, n(%)	10(20%)
Heel bone, n(%)	7(14%)
Sternum, n(%)	5(10%)
metatarsal bone, n(%)	2(4%)
Humerus, n(%)	2(4%)
other, n(%)	5(10%)
**Route of infection**	
Infection after internal fixation of the fracture	62%
Hematogenous osteomyelitis	12%
Bone adjacent tissue infection	12%
Infection after open-heart surgery	10%
Infection around hip replacement prosthesis	2%
Infection after arthroscopic knee surgery	2%
**History**	
More than 2 surgeries	52%
Combined hypertension	26%
Complicated diabetes	24%
First surgery at enrollment	14%

### Pathogenic microorganism results

#### Morphological examination

The positive rate was 32% (16/50), and all were detected by Gram staining and were consistent with the corresponding culture results (Supplemental File 1).

#### Microbial culture

**Fig. 2:**
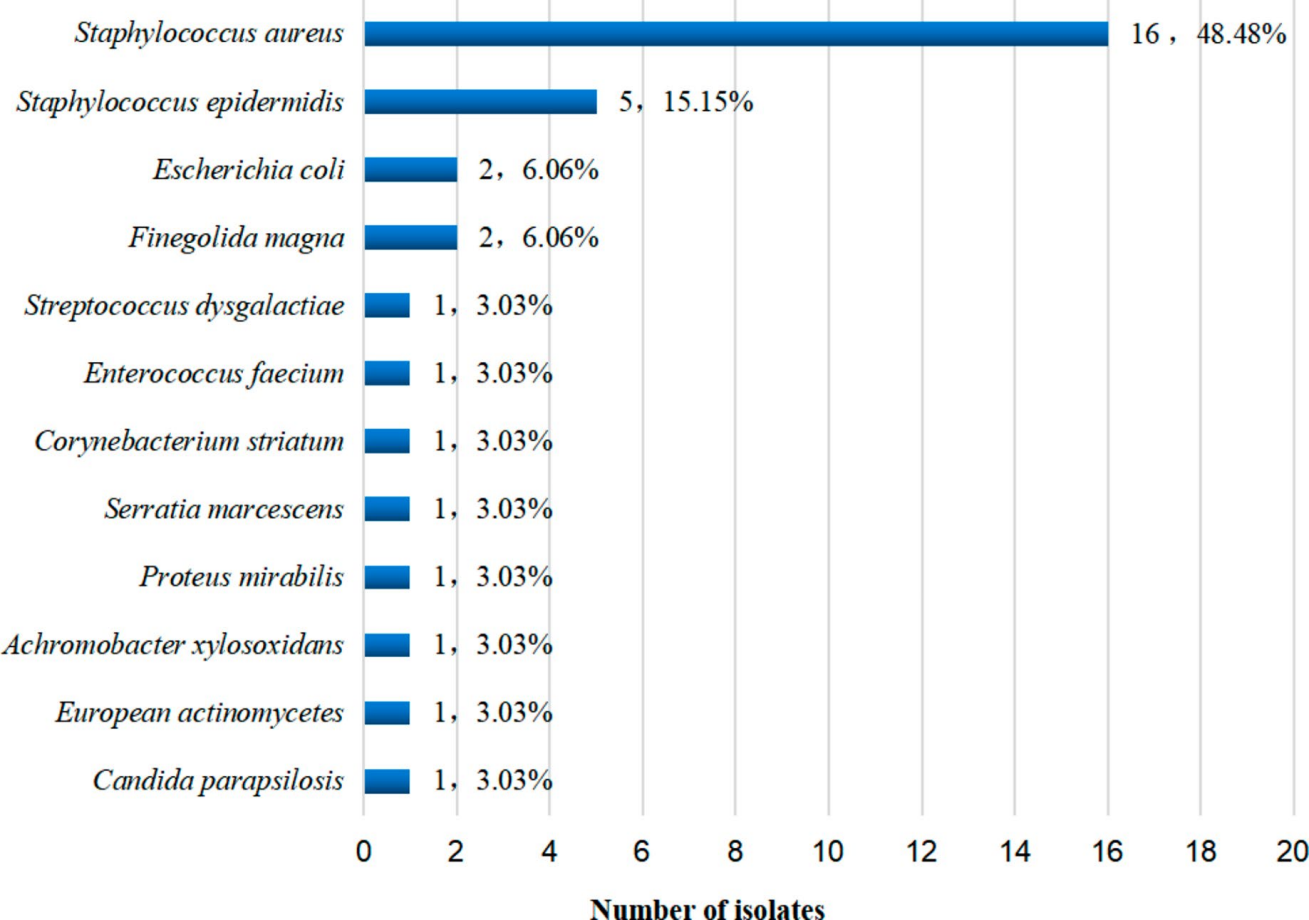
Distribution of pathogenic microorganisms detected by culture. Sixteen strains of *S.aureus*, of which 3 of them are MRSA(Methicillin Resistant Staphylococcus aureus); 5 strains of *S. epidermidis*, of which 2 of them are MRSE(Methicillin Resistant Staphylococcus epidermidis); 2 strains of *E. coli;* 2 strains of *Finegoldia magna;* 1 strain of *Streptococcus dysgalactiae*, 1 strain of *Enterococcus faecium*, 1 strain of *Corynebacterium striatum*, 1 strain of *Serratia marcescens*, 1 strain of *Proteus mirabilis*, 1 strain of *Achromobacter xylosoxidans*, 1 strain of *European actinomycetes, and 1 strain of Candida parapsilosis*

A total of 15 species and 38 microorganisms were cultured, and 12 species and 33 pathogenic microorganisms were identified by clinical diagnosis (Supplemental File 1 and Fig. 2) with a positivity rate of 56% (28/50). The top 4 detected species were *Staphylococcus aureus*, *Staphylococcus epidermidis*, *Escherichia coli*, and *Finegoldia magna*. Furthermore, mixed infections (2 or more pathogens) were found in 5 cases.

#### mNGS

**Fig. 3:**
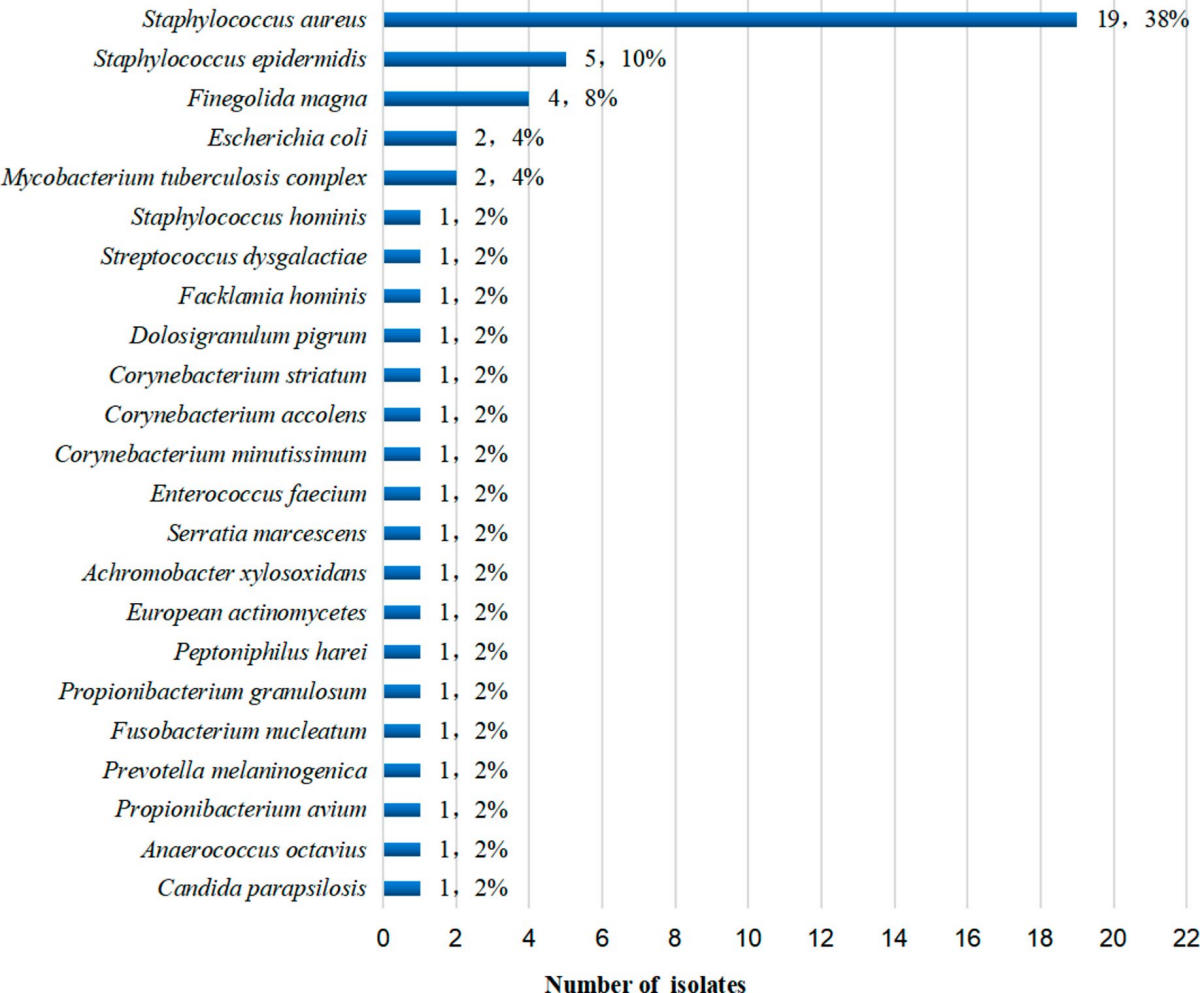
Distribution of pathogenic microorganisms detected by mNGS. Nineteen strains of *S. aureus;* 5 strains of *S. epidermidis;* 4 strains of *Finegoldia magna;* 2 strains of *E. coli;* 1 strain of *Staphylococcus hominis*, 1 strain of *Streptococcus dysgalactiae*, 1 strain of *Facklamia hominis*, 1 strain of *Dolosigranulum pigrum*, 1 strain of *Corynebacterium striatum*, 1 strain of *Corynebacterium accolens*, 1 strain of *Corynebacterium minutissimum*, 1 strain of *Enterococcus faecium*, 1 strain of *Serratia marcescens*, 1 strain of *Achromobacter xylosoxidans*, 1 strain of *European actinomycetes*, 1 strain of *Peptoniphilus harei*, 1 strain of *Propionibacterium granulosum*, 1 strain of *Fusobacterium nucleatum*, 1 strain of *Prevotella melaninogenica*, 1 strain of *Propionibacterium avium*, 1 strain of *Anaerococcus octavius;* 2 strains of *Mycobacterium tuberculosis;* 1 strain of *Candida parapsilosis*

The mNGS positivity rate was 68% (34/50). 65 microorganisms of 34 species were obtained, and a total of 24 species and 50 strains of pathogenic microorganisms with clinical significance were finally identified (Supplemental File 1 and Fig. 3) with a high detection rate of *S. aureus*, *S. epidermidis*, *E. coli*, and MTB. 11 cases (22%) were mixed infections.

### Comparison of culture and mNGS results

**Fig. 4:**
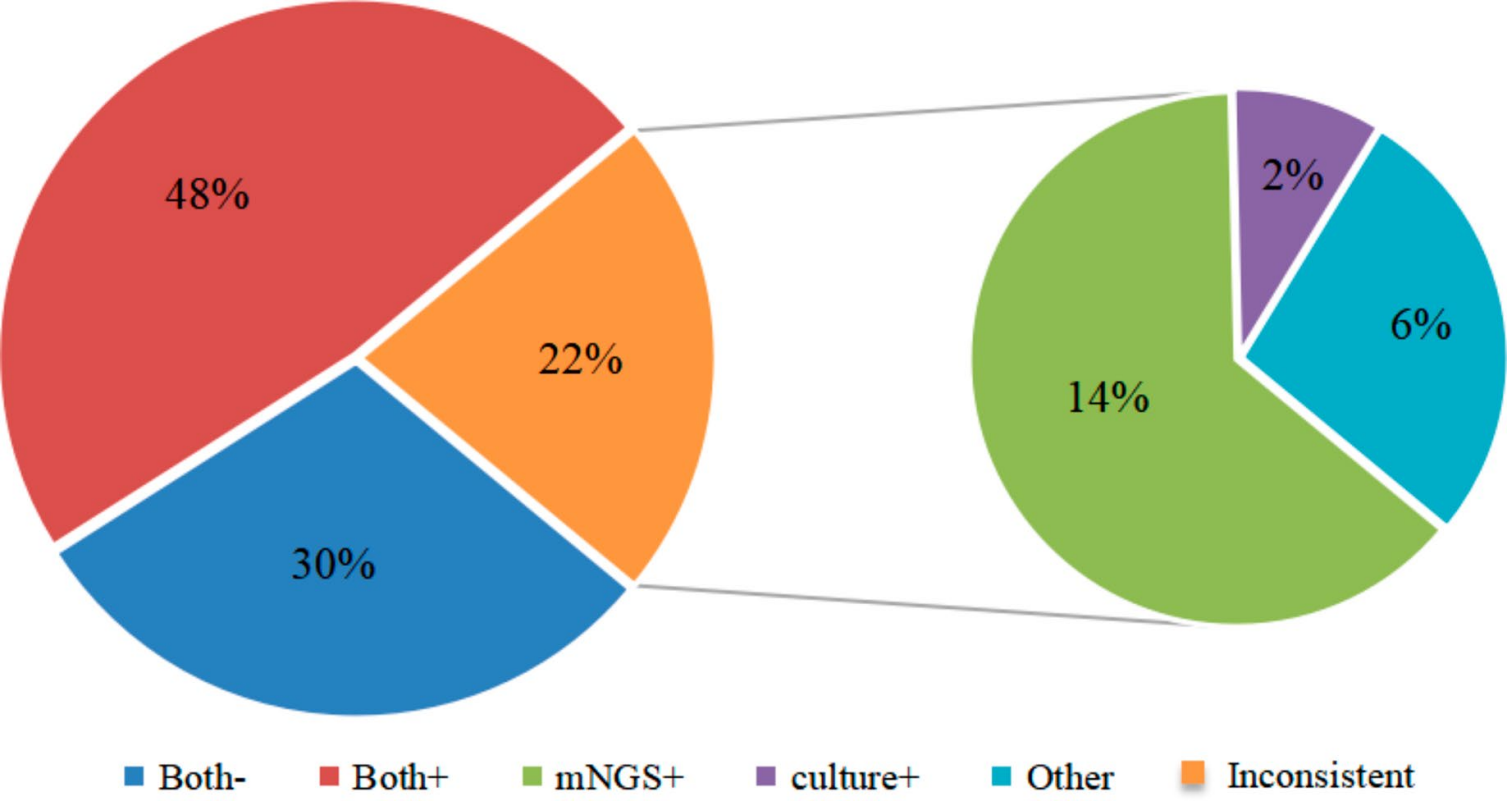
Differences between mNGS and microbial culture. Both-: both mNGS and culture results were negative; Both+: both mNGS and culture results were positive; Inconsistent: the results of mNGS and culture were not completely consistent; mNGS+: only mNGS results were positive, and culture was negative; Culture+: only culture results were positive, and mNGS was not detected; Other: both mNGS and culture results were positive, but mNGS was detected in high number

There were 39 cases where the microbial culture ultimately agreed with the mNGS results. There were 10 microbial species with more mNGS than culture, including 7 patients with negative and positive mNGS. *Proteus mirabilis* was cultured in one patient and mNGS was not detected (Fig. 4). Furthermore, 96.97% of the pathogenic bacteria in microbial culture were detected by mNGS, while 16 strains (including eight anaerobes strains) and two MTB strains were not cultured and identified. There was no statistical difference in the overall positive detection rate between mNGS and microbial culture (*P* = 0.07). The culture method detected 29 aerobic and partly anaerobic strains in 25 cases, and mNGS detected 36 strains in 27 cases. However, the difference was not statistically significant (*P* = 0.625). For the detection of anaerobic bacteria and MTB, mNGS detected a total of 13 strains in 10 cases, which was higher than the culture method (3 strains in 2 patients), with a significant difference (*P*<0.05). Furthermore, for mixed infections, 11 patients of mNGS were superior to 5 cases of culture (*P*<0.05). The comparison between the two methods for detecting different types of pathogens is shown in Table 2.

**Table 2:** Detectable pathogen count by mNGS and culture methods

Types of Pathogenic Microorganisms	mNGS	culture	*P*
Total	68%	56%	0.07
Aerobic and facultative anaerobes	54%	50%	0.625
Anaerobe and MTB	18%	4%	<0.05
Mixed infection	22%	10%	<0.05

## Discussion

An mNGS-based analysis of the composition of pathogenic microorganisms in chronic osteomyelitis was conducted, and the etiological findings contributed significantly to the efficacy of anti-infective treatments in clinical practice. Meanwhile, the limitations of etiological diagnosis were identified by comparing mNGS with the conventional microbiological detection methods currently used in laboratories. This comparison has led to improvements in conventional culture methods and the optimization of detection conditions. In some studies, the median cost of hospitalization for patients with post-traumatic osteomyelitis in China was US$10,504, with a median total hospital stay of 22 days [23]. For chronic osteomyelitis, the primary treatment principles of complete lesion removal, anti-infection, and bone reconstruction have been developed internationally [24, 25]. Notably, identifying the pathogenic microorganism of chronic osteomyelitis can shorten the patient’s disease and avoid the problem of drug resistance associated with empirical antibiotic use.

In this study, the composition ratio of pathogenic microorganisms in chronic osteomyelitis was analyzed based on the mNGS technique to compare the shortcomings of traditional methods in pathogenic diagnosis. Notably, the morphological positivity rate was only 32%, and performing a morphological examination of orthopedic specimens took more work. Such specimens require special pretreatment before smear staining, and the degree of homogenization and the ability of the testing personnel can affect the results. Additionally, Herbert Gbejuade et al. showed low sensitivity to the Gram staining method for detecting pathogens in orthopedic samples [26]. Under suitable preprocessing conditions, experienced microbiologists can perform an initial rapid mimicry. However, the smear results from most laboratories need to meet clinical needs.

### Consistency of incubation method with mNGS

The highest detection rate by culture method continued to be *S. aureus*, which is more consistent with the findings of other studies on pathogenic microorganisms of chronic osteomyelitis based on common bacterial cultures [27, 28]. Three of the 16 *S. aureus* strains had MRSA (18.75%), and most international studies had MRSA detection rates between 20% and 50% [29, 30]. Two of the five *S. epidermidis* strains were Methicillin-Resistant Staphylococcus epidermidis. The mNGS mainly identified 11 strains of anaerobes [31] and 2 strains of MTB [32, 33]. However, the overall positive detection rate did not reach statistical significance. For different types of microorganisms, the agreement between the two was better in detecting aerobic and parthenogenic anaerobic bacteria. Importantly, it showed that common bacterial cultures commonly used in microbiology laboratories could cover aerobic and partly anaerobic bacteria in chronic osteomyelitis. However, mNGS detected 6 more aerobic and partially anaerobic strains than culture. Due to the culture method being susceptible to previous antibiotic use; and chronic osteomyelitis tends to form biofilms that protect its internal bacteria from host immune cells and immune factors [34], resulting in low or negative culture positivity, mNGS is a vital tool to address these issues [9].

### Microorganisms with large statistical differences

In this study, the detection of anaerobic bacteria and Mycobacterium tuberculosis was more favorable by mNGS (*P* < 0.05). Analysis of the reasons: (i) The ideal anaerobic culture is bedside inoculation [35] since the vast majority of specimens in this study are tissues, subject to pretreatment, and the anaerobic bag culture method is challenging to maintain an anaerobic environment all the time [36], which affects the survival rate of anaerobic bacteria to some extent. (ii) Anaerobic bacteria usually promote the progression of infection together with aerobic bacteria [37]. However, the culture method only screens for microorganisms that grow luxuriantly under specific nutritional conditions. For example, staphylococci are more likely to grow than anaerobes, so certain anaerobes are missed. (iii) The detection rate of MTB by solid culture is only 45-60% [38, 39], and the long period (4–6 weeks) limits its use in early diagnosis [40]. Notably, several studies have reported the specificity of mNGS for detecting MTB to be more than 98%, and its diagnostic efficiency for extra-pulmonary TB is significantly better than that of conventional culture and XpertMTB/RIF [41, 42], which also dramatically reduces the detection time (24–48 h). The 2 cases in this study had a low number of MTB sequences detected, and although IGRA was positive, solid cultures and XpertMTB were negative. And, the mNGS is equally superior to microbial culture in mixed infections (*P* < 0.05). Furthermore, mNGS can overcome the disadvantages of the culture method, i.e., detection of Fastidious bacteria, less abundant microorganisms influenced by coexisting microorganisms, and residual nucleic acids [11], which facilitates the selection of clinical antibiotics.

### Application Value of mNGS in etiological diagnosis of chronic osteomyelitis

Anaerobic bacteria and Mycobacterium tuberculosis are key pathogens in chronic osteomyelitis infections. Chronic osteomyelitis, whether resulting from trauma, continuous infection, or hematogenous spread, often involves mixed infections of aerobic and obligate anaerobic bacteria. Tuberculous osteomyelitis is also commonly reported. However, clinical and microbiological laboratories often need to pay more attention to both due to the laborious and time-consuming culture processes and low detection rates. In this study, mNGS improved the detection rates of anaerobic bacteria and MTB in chronic osteomyelitis, and microbiological laboratories to enhance their detection conditions according to the pathogen spectrum, such as the use of anaerobic tanks or incubators, glove incubators, the rapid liquid culture of MTB, nucleic acid detection, and other methods to improve the positive detection rates. Studies have shown that the microbial culture method generally meets the diagnostic needs for aerobic and facultative anaerobic infections. Therefore, conducting mNGS tests without performing routine microbial detection in clinical practice is not recommended. Moreover, while mNGS is a valuable tool for etiological diagnosis, it currently needs more standardization regarding technical methods, data storage, bioinformatics analysis, and result interpretation [43]. Conversely, microbial culture is more cost-effective and practical, providing strain isolation and drug sensitivity results, and can be used for further research. Effective communication and collaboration between microbiology laboratories and clinicians are recommended to predict possible types of microorganisms through provisional diagnosis, followed by selecting appropriate tests such as microscopy, microbial culture, 16 S rDNA, Gene Xpert, and serological tests. Ultimately, for highly clinically suspected infections with no specific etiological diagnosis or effective conventional anti-infective treatment, mNGS can be considered in tandem with conventional etiological detection in certain patients with severe infections and patients with infections in immunocompromised to save valuable treatment time.

### Limitations

Only 50 patients were included in this study, and the laboratory is also collecting more cases of chronic osteomyelitis to analyze the relevant clinical features, conduct cohort studies, and eventually optimize the existing testing protocols in the clinical laboratory. In doing so, these can solve the critical problems in clinical diagnosis and treatment and reduce patients’ physical and psychological economic burden. Together with advances in surgery and the involvement of some new treatments [44], these can achieve better clinical results in treating chronic osteomyelitis.

### Electronic supplementary material

Below is the link to the electronic supplementary material.

Supplementary Material 1

## Data Availability

Original datasets are available in a publicly accessible repository: The raw sequence data reported in this study have been deposited in the Genome Sequence Archive (Genomics, Proteomics & Bioinformatics 2021) in National Genomics Data Center (Nucleic Acids Res 2022), China National Center for Bioinformation / Beijing Institute of Genomics, Chinese Academy of Sciences (GSA: CRA012712) that are publicly accessible at https://ngdc.cncb.ac.cn/gsa. The database used to perform the bioinformatics analysis in this study was obtained from the sequencing laboratory [WillingMed Technology (Beijing) Co., Ltd.]. The website for accessing the NCBI dataset is https://ftp.ncbi.nlm.nih.gov/genomes/refseq/.
